# Supplementary material for: Physio-fUS: a tissue-motion based method for heart and breathing rate assessment in neurofunctional ultrasound imaging
Source: eBioMedicine. 2025 Jan 31;112:105581. doi: 10.1016/j.ebiom.2025.105581 (PMC11833147; doi:10.1016/j.ebiom.2025.105581)
Supplement: Caption for Supplementary Materials [file mmc2.docx]

**Supplementary Figure 1 :** Signal processing pipeline applied to extract frame-to-frame brain tissue motion from raw ultrafast ultrasonic data. a. **In-phase and quadrature frames displayed** in logarithmic scale obtained at a framerate of 500 Hz in a mice brain at time $t_{0}$ and $t_{0+2ms}$ in a coronal plane. b. **Schematic of a radio-frequency signal** recorded in a pixel a time $t_{0}$ and $t_{0+2ms}$. c. **2D brain tissue displacement computed** the probe direction between time $t_{0}$ and $t_{0+2ms}$.

**Supplementary Figure 2 :** Diagram of the processing steps for heart and breathing rate region of interest selection.

**Supplementary Figure 3. The periodicity of pulsatility in brain tissue depends on the region in which it is averaged**.

a. **Ultrafast B-mode imaging** averaged over 200 frames at 500 Hz overlaid with 4 manually selected regions of interest (ROI).

b. **Spatial averaging of tissue velocity in the four regions of interest** over a 4-second time window. A peak detection algorithm is applied and displayed by vertical red dashed lines, showing periodicity of tissue velocity.

**Supplementary Figure 4. SVD decomposition of tissue velocity and selection of the two regions of interest for breathing and heart rate in a coronal and sagittal mouse**. a,d. **Space-time SVD decomposition** and selection of singular vectors corresponding to heart rate and breathing rate. **Selected vector is highlighted** by a red star for heart rate and a blue star for breathing rate. b,e. **Construction of two masks from selected singular vectors**. c,f. 2D **Power Doppler slice corresponding to the acquisition plane** in a coronal (Bregma -1·4mm) and a sagittal (Sagittal-median +2 mm) anaesthetized mouse.

**Supplementary Figure 5. Descriptive diagram of the step-by-step selection of two singular vectors for extraction of the breathing region of interest and the heart rate region of interest from signals used in Figure 2**. a. **The first six more energetic temporal vectors are retained**. b**. Normalised autocorrelation of time vectors** is displayed. c. A Matlab **peak detection algorithm** is applied on the extracted region of the autocorrelation (lag greater than 0.1 seconds, 500Hz), represented as a gray patch. The intensity threshold, represented by a red dashed line, is fixed to 0.3. Detected peaks are marked with a red arrow. d. **The first time position of the peaks is selected** and the relative heights of the autocorrelation are compared in a temporal window around the first peak (red patch). e. **The second time position of the peaks is selected** and the relative heights of the autocorrelation are compared in a time window around the second peak (blue patch). f. **The two singular vectors selected for heart rate and breathing rate are framed in blue and red.**

**Supplementary Figure 6. Application of the singular vector's selection process in multiple temporal windows in an anaesthetised coronal mice (Bregma 2**·**1mm), in a sagittal plane in mice and a coronal plane in a rat. a. Singular values (SV) amplitude distribution** for the 150 temporal windows of 4-sec making up the 10min acquisition. **b. Singular value selected** for heart and breathing rate extraction across temporal windows. c) d) **Time average of the region selected** for respiration (left panel) and heart rate (right panel) by the pipeline in the 150-time windows overlaid with a mouse brain Atlas at Bregma 2·1mm. e),f) **Singular value selected** for heart and breathing rate extraction across temporal windows in a sagittal plane in a mouse (left panel) and in a rat (right panel).

**Supplementary Figure 7. Additional comparison of HR and BR extracted with fUS and with electrodes in 3 mice.** For each mice measures of physiological parameters with the two approaches are overlaid in two coronal planes and a sagittal plane. HR is sampled at 1 Hz and BR at 0.5 Hz.

**Supplementary Figure 8. Additional comparison of HR and BR extracted with fUS and with electrodes in 4 rats in one coronal plane and a sagittal plane or another coronal plane.**

**Supplementary Figure 9. Monitoring of rat's heart rate using ultrafast ultrasound neuroimaging and electrodes in an experimental setup with progressive death of the animal.** a. **Superposition of heart rate assessed with electrodes and fUS** in a sagittal plane two rats that are dying due to the injection of Euthasol. b**. Quantification of the variations** of Heart rate assessed by the two approaches for the first points of the curve, a temporal region represented by the green dashed line. Curve modelling of the slope *fUS = a*Electrodes + b* for HR and BR in 2 acquisitions. Regression coefficient and coefficient of determination are computed and shown. Heart rate values higher than 400 bpm were considered as artefacts (3·6% for the top panel and 1·6% of points for the bottom one are rejected).

**Supplementary Figure 10. Schematic diagram of the implementation of real-time assessment of heart rate and respiratory rate in an ultrafast ultrasound imaging system**. a. **A 10 sec time-window is selected** (2000 frames at 500Hz). b. **Breathing rate and heart rate regions of interest are computed** with the pipeline and displayed overlaid with the mouse Atlas at bregma -2·1mm. The average time required for this initialisation is 5·3 (± 0·76 s ) seconds in mice. c. **Cardiac pulsatility signal and respiratory motion are computed** as the spatial average of the tissue velocity signal in the two previously defined regions. The time needed for this processing is 1·06 (± 0·07 s) seconds in mice. d. A **peak-detection algorithm is applied** and the mean period between peaks is displayed. e) **Diagram of the possible screen of an ultrafast scanner** in which the pipeline is implemented, allowing simultaneous acquisition of brain activity (Power-doppler middle-panel) and physiological parameters (right-hand panel). The process requires an initialisation step and a monitoring one.

**Supplementary Figure 11. Synchronised assessment of heart rate and respiratory rate during ultrasound imaging of brain activity during a sleep session in two coronal rats acquisition**. a,c**. 2D Power-Doppler images** of the coronal slice of the rat at Bregma -3·7mm (top) and Bregma 1·8mm (bottom). b,d. **Variation of the whole-brain normalised CBV** assessed by averaging the power-Doppler image over time, **with heart and breathing rate assessed with the Physio-fUS pipeline**.

**Supplementary Figure 12. Tissue velocity and heart rate in a sagittal rat freely moving in an arena.** a. **Average speed of tissue** in the heart rate region of interest during a height minute acquisition. b. Temporal zoom in two temporal regions of twenty seconds. c. Plot of the **animal's displacements in the 1m by 1m arena** during the 8-minute acquisition period. The color of the curve corresponds to the measured heart rate, a dashed line symbolises that no heart rate was measured. d. **Heart rate variation is computed** as the average time between peaks in the tissue velocity signal. In this session, the approach allowed to extract heart rate in 88% of the time points.

**Supplementary Figure 13. Synchronised functional ultrasound imaging of brain activity and heart rate assessment in human neonates**. a. The superposition of heart rate assessed with ECG and fUS in four neonates with a sampling frequency of 0.5 Hz is presented; distinct sleep phases are represented by color patches. Normalised variations of an EEG channel are plotted on the same graph. b. Bland-Altman plot of the measure of HR with reference electrodes and the Physio-fUS pipeline in N=6 neonates.

**Supplementary Table 1. Description of the number of included animals, imaging planes and their associated ethics protocol and reference publication.**

**Supplementary Table 2.** **Peak detection parameters for the extraction of tissue motion from Powerlab device and fUS imaging Data.** Intensity threshold corresponds to the minimum peak heights the function detects. Time between peaks is the minimal distance in seconds between detected peaks. The window size and step are corresponding to the time-period selected to compute the average heart and breathing rates. Std peak threshold corresponds to the threshold fixed to select time-periods in which the standard deviation of time between peaks is sufficiently high. Low and high frequency thresholds are the frequency boundaries the algorithm is tracking. In neonates data, a periodogram was used to extract heart rate.

**Supplementary Table 3. Demographic table for the preterm neonates used in the study. Sex data were collected based on the sex assigned at birth.**
